# Air pollution emissions from Chinese power plants based on the continuous emission monitoring systems network

**DOI:** 10.1038/s41597-020-00665-1

**Published:** 2020-10-05

**Authors:** Ling Tang, Xiaoda Xue, Jiabao Qu, Zhifu Mi, Xin Bo, Xiangyu Chang, Shouyang Wang, Shibei Li, Weigeng Cui, Guangxia Dong

**Affiliations:** 1 School of Economics and Management, Beihang University, Beijing, 100191 China; 2 School of Economics and Management, Beijing University of Chemical Technology, Beijing, 100029 China; 3 Appraisal Center for Environment and Engineering, Ministry of Ecology and Environment, Beijing, 100012 China; 4 The Bartlett School of Construction and Project Management, University College London, London, WC1E 7HB UK; 5 College of Resources and Environment, University of Chinese Academy of Sciences, Beijing, 100049 China; 6 State Environmental Protection Key Laboratory of Numerical Modeling for Environment Impact Assessment, Ministry of Ecology and Environment, Beijing, 100012 China; 7 School of Management, Xi’an Jiaotong University, Xi’an Shaanxi, 710049 China; 8 Academy of Mathematics and Systems Science, Chinese Academy of Sciences, Beijing, 100190 China; 9 School of Earth Science and Resources, Chang’an University, Xi’an Shaanxi, 710054 China; 10 China National Environmental Monitoring Centre, Beijing, 100012 China

**Keywords:** Environmental economics, Environmental impact

## Abstract

To meet the growing electricity demand, China’s power generation sector has become an increasingly large source of air pollutants. Specific control policymaking needs an inventory reflecting the overall, heterogeneous, time-varying features of power plant emissions. Due to the lack of comprehensive real measurements, existing inventories rely on average emission factors that suffer from many assumptions and high uncertainty. This study is the first to develop an inventory of particulate matter (PM), SO_2_ and NO_X_ emissions from power plants using systematic actual measurements monitored by China’s continuous emission monitoring systems (CEMS) network over 96–98% of the total thermal power capacity. With nationwide, source-level, real-time CEMS-monitored data, this study directly estimates emission factors and absolute emissions, avoiding the use of indirect average emission factors, thereby reducing the level of uncertainty. This dataset provides plant-level information on absolute emissions, fuel uses, generating capacities, geographic locations, etc. The dataset facilitates power emission characterization and clean air policy-making, and the CEMS-based estimation method can be employed by other countries seeking to regulate their power emissions.

## Abstract (summary)

**Table:** 

Measurement(s)	air pollution
Technology Type(s)	computational modeling technique
Sample Characteristic - Environment	fossil fuel power plant
Sample Characteristic - Location	China

Machine-accessible metadata file describing the reported data: 10.6084/m9.figshare.12813917

## Background & Summary

China has become the top power producer globally, and it had the largest share (19.5–26.7%) of global power generation from 2010 to 2018^1^. The majority (70.4–82.5% during 2010–2018) of China’s power generation came from thermal power plants that combusted coal, oil plus natural gas, biomass or other fossil energy (accounting for 60.2–73.4% of the total capacity)^2^. Accompanying with large amounts of fossil energy combustion, China’s thermal power plants have become major sources of air pollutants, emitting 5.0–23.5%, 15.7–38.7% and 19.1–51.5% of China’s anthropogenic particulate matter (PM, defined as microscopic solid or liquid matter suspended in the atmosphere)^3–6^, SO_2_^4–10^ and NO_X_^4–11^, respectively, from 2010 to 2017. These air pollutants (representing 5.9%, 23.1% and 21.5% of China’s anthropogenic PM, SO_2_ and NO_X_ emissions, respectively, in 2015^5^), through a series of physical processes and chemical reactions in the atmosphere^12^, contributed to 7.6% of China’s population-weighted PM_2.5_ (PM with an aerodynamic diameter of or below 2.5 μm) concentration as of 2015^13,14^, leading to severe haze events and human health damage nationwide.

To control power emissions, an emission inventory at high spatiotemporal resolutions is needed as the foundation for an analysis of power emission characteristics and specific policy designs^15^. There are some detailed (unit- or plant-level) inventories on air pollutant emissions from China’s power plants, such as the Global Power Emissions Database (GPED)^15^, the China Coal-fired Power Plant Emissions Database (CPED)^16^ and other bottom-up databases^17–20^. However, due to the lack of systematic real measurements, existing datasets rely on average emission factors (defined as the amount of air pollutants per unit of power generation or fuel consumption) and are subject to the following three limitations. First, average emission factors are not the results of measurements; rather, these values are proxies of broad technology classes, and the values are dependent on many assumptions and indirect parameters (e.g., pollutant contents, oxidation rates, net heating values and control technology efficiencies), which cause high uncertainty^21^. Second, average emission factors are at a quite aggregated level where they are fixed to uniform and invariable values, thereby failing to reflect the heterogeneous and time-varying features of individual power plants. Third, the available emission factors were estimated before 2012; however, China has carried out a series of mitigation measures that have brought great renovations and technological changes to Chinese power plants, such as the GB13223-2011 emissions standards implemented in 2012^22^ and the ultra-low emissions (ULE) standards promulgated in 2014^23^. Therefore, introducing direct measurements rather than using indirect average emission factors provides a promising method to reduce the uncertainty in the existing inventories.

This study is the first to develop an inventory for China’s power emissions based on systematic actual measurements^15–20^, which is named the China Emissions Accounts for Power Plants (CEAP). We introduce the data from China’s continuous emission monitoring systems (CEMS) network, i.e., the actual measurements for nationwide, real-time stack concentrations of PM, SO_2_ and NO_X_ from most of the thermal power plants in China (representing 96–98% of the total thermal capacity)^24^. The introduction of CEMS data can effectively address the three limitations in existing inventories that use the average emission factors in the following ways^15–20^. First, the CEMS-based estimation for emission factors and absolute emissions based on real emission data greatly reduces the uncertainty associated with average emission factors that are reliant on many assumptions and uncertain parameters^15^. Second, the source-level, real-time CEMS data provide rich information and improve the spatiotemporal resolutions, reflecting the heterogeneous and time-varying characteristics of power emissions^16^. Third, the CEMS data for the period of 2014–2017 are collected, and the emission factors for China’s power plants are updated, enabling an *ex post* analysis on the mitigation effects of recent clean air policies, such as GB13223-2011 and ULE standards^22,23^.

This CEAP dataset provides nationwide, detailed, dynamic PM, SO_2_ and NO_X_ emissions from China’s power plant for 2014–2017 based on comprehensive, source-level, real-time data from China’s CEMS network. In addition, the CEAP dataset encompasses rich information regarding fuel use, operating capacity, geographic distribution, etc. for each power plant. The CEAP dataset has already been employed to conduct an *ex post* analysis on the efficacy of the ULE policy in mitigating China’s power emissions^24^ and will facilitate further research on the environmental improvements and health benefits associated with the mitigation effect^14,24,25^, serve policymakers to design future clean air policies^14^, and provide implications for other countries seeking to understand and regulate their power emissions.

## Methods

### Scopes and databases

The CEAP dataset comprises all the thermal power plants operating in China, totalling 2,714 plants (or 6,267 units), from 2014 to 2017 in 26 provinces and 4 municipalities (except Hong Kong, Macao, Taiwan and Tibet; Table 1). The thermal power plants produce electricity by combusting a variety of fossil energies, which fall into 4 categories: coal, gas plus oil, biomass and others (detailed in Table 2).

**Table 1:** China’s thermal power plants in CEAP.

Year	Number of units	Number of plants	Total capacity (MW)	Unit average capacity (MW)
2014	5,943	2,583	878,240	147.78
2015	6,267	2,714	958,308	152.91
2016	6,015	2,597	983,857	163.57

**Table 2:** Fuel type descriptions.

Classification in CEAP	Fuel types in IEA
Coal	Anthracite
	Other bituminous coal
	Lignite
	Sub-bituminous coal
Gas	Natural Gas
	Natural gas liquids
Oil	Gas/diesel oil
	Crude oil
	Fuel oil
	Non-specified oil products
	Gasoline type jet fuel/Kerosene type jet fuel
	Other Kerosene
	Liquefied petroleum gases (LPG)
	Bitumen
	Petroleum coke
	Naphtha
Biomass	Primary solid biofuels
	Other liquid biofuels
	Non-specified primary biofuels and waste
	Biogases
Others	Peat
	Refinery gas
	Biogasoline
	Coal water mixture
	Blast furnace gas
	Coke oven gas
	Gas works gas
	Industrial waste
	Municipal waste (renewable)/Municipal waste (non-renewable)
	Other recovered gases

The CEAP dataset integrates two databases, i.e., the CEMS data and unit-specific information. The CEMS data—the direct, real-time measurements of stack gas concentrations of PM, SO_2_ and NO_X_ from China’s power plant stacks—are monitored by China’s CEMS network and reported to the China Ministry of Ecology and Environment (MEE; http://www.envsc.cn/). The CEMS data are recorded on a source and hourly basis. In total, the CEMS dataset covers 4,622 emission sources (i.e., power plant stacks) associated with 5,606 units (accounting for 98% of China’s thermal power capacity), 35,064 hours from 2014 to 2017, and 3 air pollutants (i.e., PM, SO_2_ and NO_X_) for each source-hour sample (Table 3). The MEE has also provided stack-specific information (regarding latitude and longitude, heights, temperature, diameter, etc.; http://permit.mee.gov.cn/).

**Table 3:** CEMS coverage of China’s thermal power plant units or stacks in CEAP.

Year	CEMS coverage		
	Number of units	Number of stacks	Coverage of total capacity
2014	5,248	3,192	96.01%
2015	5,606	3,527	97.15%
2016	5,367	3,749	95.91%
2017	5,367	4,622	95.91%

Unit-specific information is also derived from the MEE, involving activity levels (energy consumption and power generation), operating capacities, geographic allocations and pollution control equipment (particularly the types and removal efficiencies) at a yearly frequency. Due to data availability, the unit information is available only until 2016, and the activity levels for 2017 are projected following the overall trends in provincial thermal power generation between 2016 and 2017 (which are available in the *China Energy Statistical Yearbooks*^26^), under the assumption that new units constructed in 2017 have the same structures of installed capacities, energy uses and regions as those of the existing units in 2016.

With a combination of the two datasets, the CEAP dataset provides nationwide, plant-level, dynamic PM, SO_2_ and NO_X_ emissions from China’s thermal power plants from 2014 to 2017. Relative to existing inventories, the CEAP dataset is innovative in that it incorporates comprehensive real CEMS-measured emission data, avoiding the use of average emission factors and the associated operational assumptions and uncertain parameters.

### Pre-processing of CEMS data

We have been exclusively granted access to the data from China’s CEMS network. Generally, the CEMS consists of a sampling system (for filtering and sampling flue gas), an online analytical component (for monitoring flue gas parameters, particularly emission concentrations) and a data processing system (for collecting, processing and reporting monitoring data)^27,28^. According to the GB13223-2003 regulation^29^, the CEMS network should cover all power plant furnaces that burn coal (except stoker and spreader stoker) and oil and generate >65 tons of steam each hour, as well as those that burn pulverized coal and gas. Thus, some power plants have not yet been incorporated into the CEMS network (accounting for 3–4% of the total thermal power capacity from 2014 to 2017) because their furnaces did not meet the requirements necessary to install a CEMS. For the power plants outside the CEMS network, we assume their stack concentrations are similar to the averages of the units with similar fuel types and similar regions within the CEMS network.

To guarantee the reliability of CEMS data, China’s government has made great efforts in developing specific regulations and technical guidelines for power plants and local entities to follow and supervise, respectively^24,28,30–32^. These official documents elaborate on all the processes required to regulate the CEMS network, including not only CEMS installation, operation, inspection, maintenance and repair but also CEMS data collection, processing, reporting, analysis and storage^28,32,33^. Since 2014, all state-monitored companies have been mandated to report their CEMS data to the local governments through a series of online platforms for different provinces (listed in Supplementary Table 1). Local entities have random onsite inspections to check the truthfulness of the reported results on at least a quarterly basis^23,24,28,32,34^; this system enables a comparison of CEMS data across different firms to explore potential outliers and abnormalities and prevent data manipulation^28,35^. Then, the governments release the inspection results to the public through the same online platforms (listed in Supplementary Table 1)^24,36,37^. Severe financial penalties and criminal punishments can be imposed on firms that adopt data manipulation (in terms of deleting, distorting and forging CEMS data, for example)^38,39^.

The malfunction of CEMS monitors may also introduce large uncertainty to CEMS data during the processes of operation (indication errors, span drift, zero drift, etc.), maintenance (particularly the failure to perform calibration and reference tests) and data reporting (invalid data communication, data missing, etc.)^24,28^. Accordingly, each power plant is required to make at least one A-, B- and C-grade overhaul for 32–80, 14–50 and 9–30 days per 4–6, 2–3 and 1 year(s), respectively, as well as one D-grade overhaul (if needed) for 5–15 days per year, to check, maintain and upgrade its technologies, thereby reducing measurement uncertainty^40^. During these overhauls, CEMS operators conduct CEMS calibration (i.e., zero and span calibration), maintenance procedures (e.g., examining and cleaning major CEMS components and replacing or upgrading parts, if necessary, such as optical lens, filter and sampling meter) and a reference test (i.e., relative accuracy test audit). Furthermore, third-party operators examine CEMS operation and maintenance routines, to guarantee standardized CEMS operation and facilitate improvement in CEMS data accuracy^27,28,31^. All the related activities should be documented according to standardized requirement contents^27,28^. Even with the aforementioned efforts, there is still a small proportion of nulls and outliers in the CEMS database, which represent 1% and 0.1% of the total operating hours, respectively, from 2014 to 2017. We treat these samples seriously by following the relevant official documents, which have been released by China’s government. Table 4 provides the treatment methods for nulls or zeros, which can be divided into 3 types based on duration. On the one hand, we consider nulls and/or zeros that span at least 5 successive days as a downtime or overhaul and omit them in the estimation, according to the regulation^27^. On the other hand, missing data lasting < 5 day(s) are treated as outliers (i.e., impossible values in operation) and processed in two different ways: the nulls and/or zeros successive for > 24 hours are assumed around the valid values near the time and set to the monthly averages^27^:

1 $${\hat{C}}_{s,i,y,m,h}={\bar{C}}_{s,i,y,m,\bullet }$$

where ${C}_{s,i,y,m,h}$ denotes the stack gas concentrations of pollutant *s* emitted by unit *i* for year *y*, month *m* and hour *h* (i.e., the actual measurements monitored by the CEMS network), defined as the amount of pollutants per unit of emitted stack gas (g m^−3^)^41,42^; ${\widehat{C}}_{s,i,y,m,h}$ is the imputation for the missing data ${C}_{s,i,y,m,h}$; ${\bar{C}}_{s,i,y,m,.}$ is the mean of the hourly valid values for the same pollutant, unit, year and month as ${C}_{s,i,y,m,h}$. In contrast, the missing data for 1–24 hour(s) are interpolated with the arithmetic averages of the two nearest valid points before and after them^27,43^:

2 $${\widehat{C}}_{s,i,y,m,h}=\frac{{C}_{s,i,y,m,h-l}+{C}_{s,i,y,m,h+q}}{2}$$

where ${C}_{s,i,y,m,h-l}$ and ${C}_{s,i,y,m,h{\rm{+}}q}$ represent the nearest last known measurements (*l* hour(s) before) and next known measurements (*q* hour(s) after), respectively, for the missing data ${C}_{s,i,y,m,h}$, namely, the series data ${C}_{s,i,y,m,h-l+1}$,…, ${C}_{s,i,y,m,h}$,…, ${C}_{s,i,y,m,h+q-1}$ are all missing values. Furthermore, we treat the measurements that are out of the measurement ranges of CEMS instruments (outside of which the data are unreliable^30,44^; detailed in Supplementary Table 2) as abnormal data and process them in a similar way to nulls according to the official regulation^27^.

**Table 4:** Treatment methods for nulls and the relevant official documents.

Type	Descriptions	Treatment method	Supporting official documents
1	Successive nulls for >5 days	Consider them as downtime for maintenance and omit them in emission.	a. According to the regulation^40^, a power plant should go through at least one long maintenance shutdown per year, with one lasting for at least 5 days.
			b. The estimated downtime (corresponding to successive nulls for at least 5 days) accounted for 17.11% of the time for 2015, which are generally consistent with the official statistics (19.41%)^9^ (considering that 3–4% of plants do not have CEMS).
2	Successive nulls for 1–5 days	Assume them around the levels of valid values near the time (in terms of monthly averages).	Chinese government published *Specifications for Continuous Emissions Monitoring of Flue Gas Emitted from Stationary Sources* (HJ/T 75-2007)^27^. It suggests no interpolation for successive missing data of emission concentrations lasting for above 24 hours during operation, assuming them at similar levels to the points near the time and not to largely deviate from the average values.
3	Nulls lasting for 1–24 hour(s) (involving non-successive nulls)	Set them to the arithmetic mean of the two nearest valid points before and after them.	The guideline (HJ/T 75-2007)^27^ suggests setting missing data lasting for 1–24 hour(s) to the arithmetic mean of the two nearest valid points before and after them.

### CEMS-based estimation of emission factors and absolute emissions

The introduction of real CEMS-monitored measurements provides a direct estimation for emission factors on a source and hourly basis, avoiding the use of average emission factors with many assumptions and uncertain parameters^17,42,44^.

3 $$E{F}_{s,i,y,m,h}={C}_{s,i,y,m,h}{V}_{i,y}$$

In Eq. (3), $E{F}_{s,i,y,m,h}$ indicates the emission factor, defined as the amount of emissions per unit of fuel use (in g kg^−1^ for solid or liquid fuel and in g m^−3^ for gas fuel), and ${V}_{i,y}$ is the theoretical flue gas rate, defined as the expected volume of flue gas per unit of fuel use under standard production conditions (m^3^ kg^−1^ for solid or liquid fuel and m^3^ m^−3^ for gas fuel)^42^, which was estimated by the China Pollution Source Census (2011)^45^ based on sufficient field measurements (detailed in Table 5). Based on Eq. (3), abated emission factors can be directly obtained even without the use of removal efficiencies and the relevant parameters, because CEMS monitors the gas concentrations at stacks after the effect of control equipment (if any).

**Table 5:** Theoretical flue gas rate.

Fuel type	Boiler	Unit capacity (MW)	CPSC value ^a^ (m^3^ ton^-1^)	CEMS-based value ^b^ (m^3^ ton^-1^)	Uncertainty ranges ^b^	*t*-test ^c^
Coal	Pulverized coal-fired boiler	> = 750	8,271	8,376	±5.73%	P = 0.67, n = 48
		450~749	10,150	10,690	±4.75%	P = 0.04, n = 332
	Pulverized coal-fired boiler & Circulating fluidized bed boiler	250~449	9,713	9,790	±3.08%	P = 0.62, n = 541
		150~249	9,305	9,806	±5.61%	P = 0.08, n = 113
		75~149	8,178	8,043	±6.56%	P = 0.62, n = 58
		35~74	7,558	8,030	±6.87%	P = 0.10, n = 57
		20~34	7,729	8,038	±6.71%	P = 0.27, n = 77
	Pulverized coal-fired boiler, circulating fluidized bed boiler & stoker-fired boiler	9~19	7,958	8,452	±4.88%	P = 0.02, n = 83
Bituminous coal	Stoker-fired boiler	< = 8	10,290	13,494	±9.21%	—
	Pulverized coal-fired boiler		9,186			
	Circulating fluidized bed boiler		9,415			
Anthracite	Stoker-fired boiler		10,197			
	Circulating fluidized bed boiler		11,034			
Lignite	Stoker-fired boiler		5,915			
	Pulverized coal-fired boiler		5,915			
Coal gangue	Circulating fluidized bed boiler	—	4,806	6,718	±10.07%	P < 0.00, n = 43
Solid waste	Incinerator	—	6,722	—	—	—
Solid waste & Coal	Incinerator	—	7,774	—	—	—
Gas	Turbine	—	24.55	24.9	±9.91%	P = 0.79, n = 21
Oil	Boiler & Turbine	—	11,152	—	—	—
Petroleum coke	Circulating fluidized bed boiler	—	11,665	—	—	—

Notes: ^a^The values are derived from the China Pollution Source Census (CPSC) (2011) and used in our estimation; ^b^The results are estimated using the CEMS-monitored samples; ^c^A single-sample two- tailed t-test is conducted for each type with the null hypothesis that the mean CEMS-monitored flue gas rates deviate from the CPSC value.

Notably, recent clean air policies (particularly different emissions standards) target stack concentrations, such that a large proportion of missing data exist regarding other measurements (particularly flue gas rates, with missing data accounting for 34.62%, 31.91%, 29.97% and 42.96% of the total samples in 2014, 2015, 2016 and 2017, respectively). Accordingly, we introduce theoretical flue gas rates into the estimation to avoid significant underestimation of the actual volume when there are too many missing data values^46^. In addition, the adoption of theoretical flue gas rates can address flue gas leakage, a common problem in power plants that greatly distorts the real flue gas volume^46^. The theoretical flue gas rates are derived from the China Pollution Source Census, with values varying across operating capacities, fuel types and boiler types^42,45^. Thus, the actual volume of flue gas is computed in terms of the theoretical flue gas rate times actual fuel consumption.

The absolute emissions of PM, SO_2_ and NO_X_ from individual power plants can be estimated in terms of the emission factors times the activity levels^21^:

4 $${E}_{s,i,y,m}=E{F}_{s,i,y,m}{A}_{i,y,m}$$

where ${E}_{s,i,y,m}$ represents the air pollution emissions (g); and ${A}_{i,y,m}$ is the activity data, i.e., the amount of fuel use (kg for solid or liquid fuel and m^3^ for gas fuel). In the CEAP dataset, power plant emissions are estimated on a monthly basis (the smallest scale for activity data), in which the yearly unit-level activity data are allocated at the monthly scale using the monthly province-level thermal power generation as weights^16^:

5 $${A}_{i,y,m}=\frac{{F}_{{p}_{i},y,m}}{{\sum }_{m=1}^{12}{F}_{{p}_{i},y,m}}{A}_{i,y}$$

where ${F}_{{p}_{i},y,m}$ denotes the thermal power generation by province *P*_*i*_, which is obtained from the *Chinese Energy Statistics* *Yearbook*^26^, and ${p}_{i}$ indicates the province where unit *i* is located.

## Data Records

A total of 12 data records (emissions and plant/unit information inventories) are contained in the CEAP dataset, which have been uploaded to public repository figshare^47^. Of these

- 4 are emission inventories for China’s power plants (2014–2017) [“CEAP-Absolute emissions, 2014–2017”];
- 4 are stack gas concentration inventories for China’s power plants (2014–2017) [“CEAP- Stack gas concentrations, 2014–2017”];
- 4 are summary descriptions for China’s power plants (2014–2017) [“CEAP-Summary descriptions, 2014–2017”].

**Fig. 1:**
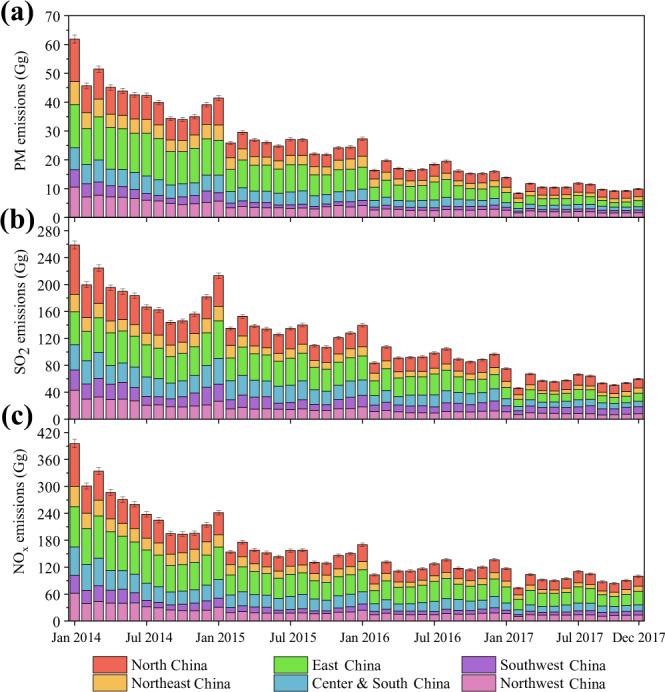
Estimated power emissions in China from 2014 to 2017. (**a**–**c**), Monthly estimates for the total and regional (coloured bars) emissions (Gg) of PM (**a**), SO_2_ (**b**) and NO_X_ (**c**) from Chinese power plants. The error bars indicate the uncertainty ranges.

The CEAP dataset introduces systematic real measurements by China’s CEMS network to directly estimate the PM, SO_2_ and NO_X_ emissions from China’s power plants during 2014–2017 (Fig. 1). In particular, the dataset provides plant-level information about absolute emissions, fuel uses, generating capacities and geographic allocations for 2,583, 2,714, 2,596 and 2,596 power plants from 2014 to 2017, respectively. In addition, the CEAP dataset presents dynamic stack concentrations by region and fuel type and describes the overall structures of operating units, capacities, ages, emission factors, emissions and CEMS coverage for China’s thermal power plants.

## Technical Validation

### Uncertainties

The CEMS-based estimates are subject to uncertainties arising from volatilities in the CEMS data, the introduction of theoretical flue gas rates and the projection of activity data. Thus, uncertainty analyses are performed to verify the robustness of our estimates. Generally, the uncertainty analysis on each examined model variable or parameter (emission concentrations, theoretical flue gas rates or activity data) includes five main steps: (a) estimate the probability distributions by fitting data with an given distribution as the input of the Monte Carlo approach; (b) generate random values based on the probability distributions via Monte Carlo simulation; (c) put the random values into Eqs. (3–5) to replace the original values and obtain a new set of estimates for emission factors and total emissions; (d) repeat steps (b) and (c) 10,000 times and obtain 10,000 sets of results^16,17,48,49^; and (e) yield the uncertainty ranges of our estimates in terms of 2 standard deviations of the new 10,000 set of results^21^. Table 6 reports the related results and reveals that the uncertainties can be controlled within a small range (i.e., ±9.03% and ±2.47% for emission factors and absolute emissions, respectively).

**Table 6:** Uncertainty ranges of the estimated emission factors and absolute emissions.

Factors leading to uncertainties	Uncertainty ranges	
	Emission factor	Absolute emissions
CEMS data	±8.65%	±1.09%
Theoretical flow gas rates	±6.90%	±0.23%
Activity data for 2017		±0.03%

### Uncertainties in CEMS data

The volatility in stack gas concentrations (the key model inputs in our estimation) should be considered in the uncertainty analysis^42^. As the hourly CEMS measurements are recorded as an average over an hour time period, the associated volatility well reflects real variability in the emissions (as power demand rises and falls throughout the day, for example)^32^. We assume normal distributions for stack concentrations for each unit on a monthly basis and then draw the related parameters of distributions (e.g., the mean and the standard deviation) through data fitting based on the associated daily averages of the CEMS measurements^50,51^. For a unit without CEMS, the bootstrap method is used to select samples from the units of the same fuel type and the same region in the CEMS network at an equal probability. Then, the Monte Carlo simulation is performed to generate random stack concentrations based on the associated distributions^17,42^. With 10,000 simulations, the uncertainty ranges of the estimates are assessed to be small, i.e., ±8.65% and ±1.09% for the emission factors and absolute emissions, respectively.

Measurement uncertainties lead to a certain level of CEMS-monitored stack concentration deviations^28^. According to the official regulation^27^, a qualified CEMS instrument should control the error tolerance within ±15%, ±5% and ±5% for PM, SO_2_ and NO_X_ concentrations, respectively. Accordingly, we assume uniform distributions within the allowed tolerance ranges for all stack concentrations on the hourly, unit and pollutant basis. Then, random stack concentrations are generated using the Monte Carlo technique and put into Eq. (3) replacing the associated original values. A total of 10,000 simulations are run to estimate the uncertainty ranges of our estimates (in terms of 2 standard deviations). The results show that the final uncertainties fall within ±10.38% for emission factors and ±0.59% for total emissions.

### Uncertainties in theoretical flue gas rates

Given that a large proportion of measurements of actual flue gas rates are missing in CEMS data (29.97–42.96% from 2014 to 2017), we introduce theoretical flue gas rates (fourth column of Table 5) in the estimation. Even though this method can prevent significant underestimations and flue gas leakage, uncertainties might arise due to the heterogeneity across units in factors such as technologies, operational situations and feedstocks. We assess the uncertainty ranges of flue gas rates (defined as the lower and upper bounds of a 95% confidence interval around the central estimates^16,48^; six column) using the real samples in the CEMS database for 1,373 units that have different unit capacities, fuel types and boiler types and are located throughout mainland China (fifth column). A single-sample two-tailed *t*-test is conducted, and the results (last column) indicate that the mean CEMS-monitored flue gas rates (fifth column) are at similar levels to the theoretical values that we used (fourth column). In the uncertainty analysis, Monte Carlo simulation is conducted to produce random flue gas rates following a uniform distribution on the associated uncertainty ranges^48,52^. For the unit types without uncertainty ranges (e.g., those burning solid waste, oil and petroleum coke), the largest range (i.e., ±10.07%) is employed. Relying on 10,000 simulations, the results show that uncertainty ranges can be well controlled within ±6.90% and ±0.23% for the emission factors and absolute emissions, respectively.

### Uncertainties in activity data

The unit-specific activity data are available only up to 2016, and the 2017 values are projected using the monthly provincial data for 2017. This approach assumes that the growth rates in the activity levels of different units in a province are uniform from 2016 to 2017, which somewhat contradicts reality and brings about uncertainties. To assess such uncertainties, a bootstrap method is used to generate 10,000 samples of the growth rates from the previous values from 2014 to 2016, and statistical analysis is employed to fit these samples in a normal distribution. The Monte Carlo simulation is performed to generate random growth rates and thence the growth of activity levels from 2016 to 2017 for individual units, and the total provincial growth is allocated into each unit using the random growth as weights. With 10,000 simulations, the uncertainty range of total emissions is estimated to be quite small (within ±0.03%).

### Comparison with existing databases

**Fig. 2:**
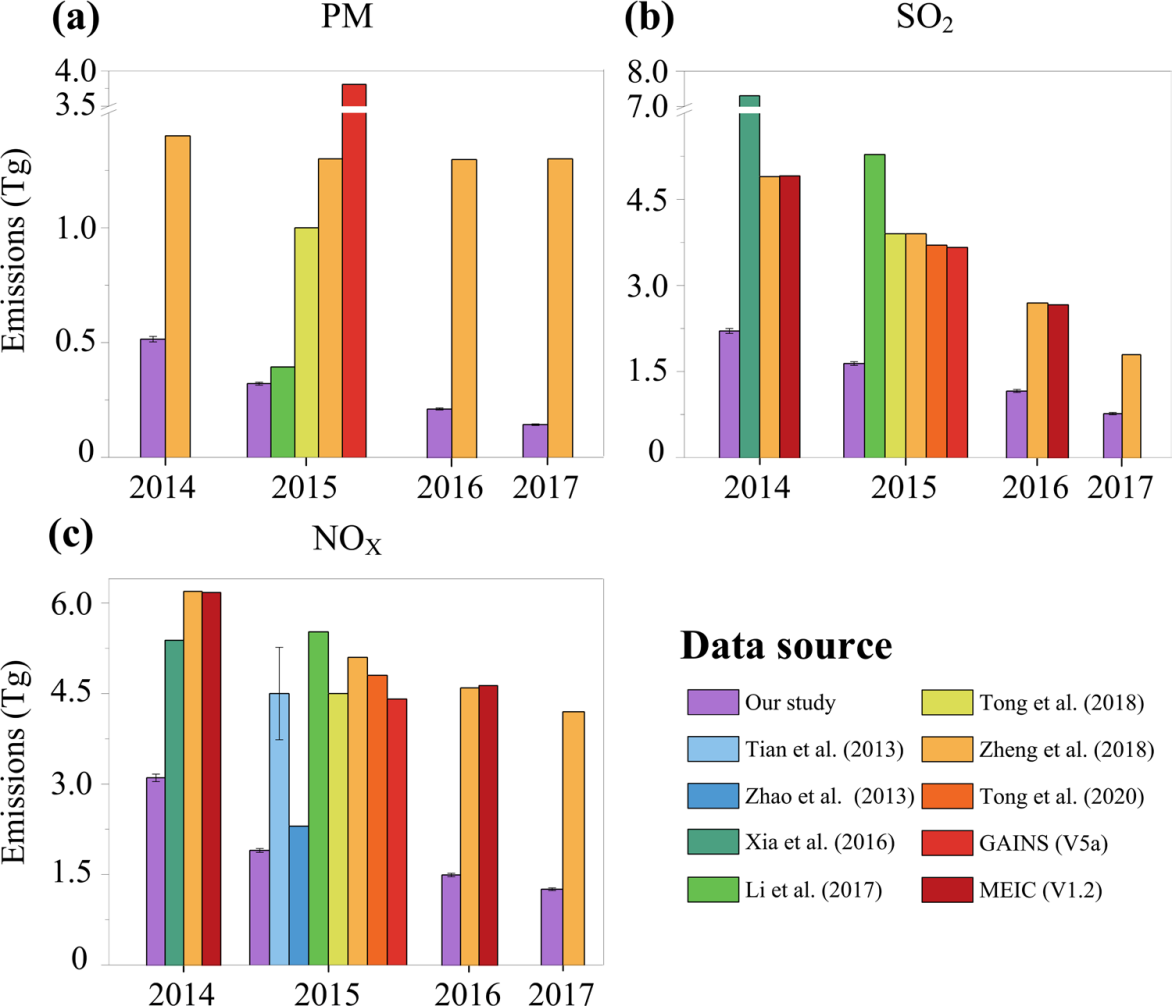
Comparison of estimated power emissions in China from 2014 to 2017. (**a**–**c**), The estimated Chinese power emissions (Tg) for PM (**a**) SO_2_ (**b**) and NO_X_ (**c**) in our database (purple bars) and in existing databases (Refs.^5,10,11,20,53–55^; the Greenhouse Gas and Air Pollution Interactions and Synergies database (GAINS) (https://gains.iiasa.ac.at/models/gains_models3.html); the Multi-resolution Emission Inventory for China (MEIC) (http://meicmodel.org/); non-purple bars). The error bars indicate the associated uncertainty ranges.

We compare our estimates with existing databases, finding that our estimates of Chinese power emissions (using the real CEMS measurements for 2014–2017; purple bars in Fig. 2) are 18.62–91.86%, 54.98–69.77% and 17.55–67.76% below previous estimates (based on average emission factors that were evaluated up to 2012 without considering the recent mitigation effect particularly attributable to the ULE standards policy promulgated in 2014) for PM, SO_2_ and NO_X_, respectively. Furthermore, using the detailed measurements on the source and hour basis, the uncertainty of our estimates can be controlled at a relatively low level (error bars).

### Limitations and future work

The CEAP dataset can be improved and extended from the following perspectives. First, some power plants have not yet been incorporated into the CEMS network, which account for an average of 3.8% of the total thermal capacity for 2014–2017. Therefore, collecting and incorporating these samples is needed to extend the CEAP dataset. Second, apart from air pollutants from power plants, the CEMS network monitors both air and water pollutants from various industries, totalling over 30,000 emission sources. Based on these data, the CEAP database can be extended into multisector datasets for both air and water pollutants in the future. Third, due to the data availability, the estimation does not use high-frequency information about activity data, such that CEMS data majorly drive the power emissions on a monthly scale. Future research involves incorporating hourly operational data (especially fuel consumption and flue gas rates) for each unit to improve the reliability of emissions estimates. Fourth, although great efforts have been made to guarantee the reliability of CEMS data, serious verification works (such as aerial concentration measurements) are still needed to check the data quality of the CEMS system^41^.

## Supplementary information

Supplementary Table 1

Supplementary Table 2

## Data Availability

There is no custom code in the generation of the CEAP dataset. In this study, Microsoft Excel is employed to process all the data and Origin is used to draw the figures. Three model inputs have been used in the construction of this dataset, i.e., the measurements from China’s CEMS network, theoretical flue gas rates and activity data. First, the CEMS-monitored data are released by the Ministry of Ecology and Environment of China through online platforms for different provinces, and we have documented all the links to these platforms in Supplementary Table 1. Second, theoretical flue gas rates are available in Table 5. Third, activity data are exclusively offered by the Ministry of Ecology and Environment of China.
